# Decomposition of regional convergence in population aging across Europe

**DOI:** 10.1186/s41118-017-0018-2

**Published:** 2017-05-10

**Authors:** Ilya Kashnitsky, Joop de Beer, Leo van Wissen

**Affiliations:** 1 Netherlands Interdisciplinary Demographic Institute, University of Groningen, Groningen, The Netherlands; 2 National Research University Higher School of Economics, Moscow, Russia

**Keywords:** Beta convergence, Population aging, Demographic decomposition, Population structures, Cohort turnover, Migration at working ages, Mortality at working ages, Total support ratio, NUTS-2

## Abstract

In the face of rapidly aging population, decreasing regional inequalities in population composition is one of the regional cohesion goals of the European Union. To our knowledge, no explicit quantification of the changes in regional population aging differentiation exist. We investigate how regional differences in population aging developed over the last decade and how they are likely to evolve in the coming three decades, and we examine how demographic components of population growth contribute to the process. We use the beta-convergence approach to test whether regions are moving towards a common level of population aging. The change in population composition is decomposed into the separate effects of changes in the size of the non-working-age population and of the working-age population. The latter changes are further decomposed into the effects of cohort turnover, migration at working ages, and mortality at working ages. European Nomenclature of Territorial Units for Statistics (NUTS)-2 regions experienced notable convergence in population aging during the period 2003–2012 and are expected to experience further convergence in the coming three decades. Convergence in aging mainly depends on changes in the population structure of East-European regions. Cohort turnover plays the major role in promoting convergence. Differences in mortality at working ages, though quite moderate themselves, have a significant cumulative effect. The projections show that when it is assumed that net migration flows at working ages are converging across European regions, this will not contribute to convergence of population aging. The beta-convergence approach proves useful to examine regional variations in population aging across Europe.

## Introduction

Population aging is the most evident demographic challenge of European countries and regions. The unprecedented increase in the share of the elderly population raises concerns about the sustainability of social and economic developments (Bloom et al. 2015; Feldstein 2006). The sharp increase in the proportion of the elderly dependent population is expected to have a significant negative impact on pension systems (Ediev 2013; Gruber and Wise 2009; Hammer and Prskawetz 2013), social and health care (Mahon and Millar 2014), and public and personal transfers towards the elderly (Dukhovnov and Zagheni 2015; Lee and Mason 2010).

Differences in the past and current developments of demographic structures contribute to substantial spatial variation of aging across European countries (Diaconu 2015) and across regions (Gregory and Patuelli 2015). Regional policies in European Union aim to reduce variation in all aspects that can influence differentiation in the quality of life, including demographic developments (European Commission 2015; Giannakouris 2008). According to the European Commission’s logic, convergence in aging is desirable because it will contribute to the reduction in regional life quality disproportions.

In this paper, we apply the widely used concept of beta convergence to study how relative differences in aging evolve (Barro 1991; Barro et al. 1991; Baumol 1986). Beta convergence utilizes linear regression approach to check the relationship between the growth and the initial distribution: if regions at the bottom of the initial distribution experience faster growth, then the variance of the distribution reduces by the end of the modeling period. To our knowledge, no other paper has explicitly analyzed population aging using the convergence research framework. Lacking any prior empirical evidence on the matter, one can distinguish two contrasting hypotheses about the possible developments of the regional differences in population aging. First, it seems reasonable to expect convergence in aging at the end of the demographic transition in Europe: European countries move along the demographic transition path with varying timing and pace, and the differences should diminish by the end of the process when populations approach the post-transitional replacement regime. Alternatively, the process of urbanization is likely to contribute to a divergent pattern of aging: urbanized regions tend to attract population at working ages, while rural regions are left with a higher proportion of people out of the labor market.

In this paper, we examine the first hypothesis. For this purpose, we analyze how regional differences in aging have changed over the period 2003–2012. In addition, we examine whether current trends in regional variation in aging will continue. For this reason, we examine Eurostat regional population projections for the upcoming three decades. In order to examine to what extent policy measures could be effective in promoting convergence in population aging, we assess the causes of changes in the working-age population: migration, mortality, and cohort turnover. Cohort turnover is defined by the difference between the numbers of young people entering and older people leaving the working ages. To the extent that cohort turnover affects convergence in aging, there is little room for policy options as the impact of cohort turnover can only be affected in the long run. To the extent that mortality affects convergence in aging, one main question is whether convergence in mortality would lead to convergence in aging. To the extent that migration affects convergence in aging, policy makers may aim to affect the direction of migration flows between regions and countries.

We identify the role of demographic components that cause changes in the ratio of the working-age to the non-working-age population (*total support ratio* (TSR)), thus influencing convergence in aging. For that reason, we decompose the convergence in TSR into the effects of changes in the non-working-age population and changes in the working-age population. The latter is further decomposed into the effects of cohort turnover, migration at working ages, and mortality at working ages. Finally, we examine the time differences of convergence in TSR during the observed and projected parts of the study period. The temporal decomposition of convergence in aging helps to identify the turning points in the recent development of regional differences in population structures and examine the possible future development.

## Demographic transition and convergence in aging

The demographic development after the baby boom is characterized by accelerating population aging, as the relatively large cohorts of the baby boom come out of working ages, and below-replacement fertility does not provide equally large successive cohorts (Lee 2003). Thus, it seems reasonable to expect convergence in aging at the end of the demographic transition in Europe: European countries move along the demographic transition path with varying timing and pace, and the differences should diminish by the end of the process (Coleman, 2002). For example, as Dudley Kirk points out (Kirk 1996, p. 366), similarities in demographic transition made United Nations and World Bank base their population forecasts on the assumption of a standard transition. Though, different timing of the second demographic transition due to cultural and behavioral variability (Lesthaeghe 2010) may affect the speed of convergence in aging considerably. Thus, one important question is whether the variability in population aging does or does not lead to convergence in aging at the regional level in Europe and whether future changes may be different from recent trends. We expect that cohort turnover, which reflects the existing disproportions in population structures, will lead to convergence in aging, but it is less obvious what will be the effect of mortality and migration.

In this paper, we use the methodological concept of beta convergence to test if the variation in aging across European regions has increased or decreased. This method was originally developed in the economic literature to study income inequalities (Barro 1991; Barro et al. 1991; Baumol 1986). The method was rarely applied to demographic data before and, to our knowledge, was never used to analyze the development of regional differences in the population age composition. Previous demographic papers used convergence analysis techniques to study spatio-temporal regularities in mortality (Edwards 2011; Edwards and Tuljapurkar 2005; Goesling and Firebaugh 2004; Janssen et al. 2016; Neumayer 2004; Richardson et al. 2014; Tuljapurkar and Edwards 2011), fertility (Dorius 2008; Wilson 2011), and migration (Barro and Sala-i-Martin 2003; Huber and Tondl 2012; Kubis and Schneider 2015; Ozgen et al. 2010).

With the use of convergence analysis, we investigate whether regional differences in aging increase or decrease over time in Europe. Beta convergence occurs when regions which were less aged at the beginning of the study period experience stronger population aging than the regions that were initially more aged. If there is beta convergence, the model predicts that all regions would reach the steady-state level of population aging in the future. If the condition is not satisfied, the modeling shows that the regions experience divergence, and there is no reason to expect a reduction in inequality.

## Data and methods

### Data

This paper uses Eurostat data on population structure (Eurostat 2015d) and mortality records by 1-year age groups regions of EU28^1^ for the period 2003–2012 (Eurostat 2015a). The data are aggregated at the NUTS-2 level, version of 2010 (Eurostat 2015c); NUTS means Nomenclature of Territorial Units for Statistics. At the moment of data acquisition (March 2015), mortality records covered the period up to 2012. For the majority of regions, data on population structure are available since 2003. Hence, the availability of data limited the observed study period to 2003–2012. We also used Eurostat regional projections (Eurostat 2015b) for three more decades, 2013–2042.

For some regions, data were partially missing. Due to the changes in administrative division at the NUTS-2 level, there were no data for all five regions of Denmark before 2007 (Kashnitsky 2017) and two regions in the eastern part of German, Chemnitz (DED4) and Leipzig (DED5) before 2006. Furthermore, mortality data were missing for Ireland in 2012, and population structure data were missing for Slovenia in 2003–2004. We reconstructed the missings using the data from national statistical offices.

Exploratory data analysis showed inconsistency of population estimates for the regions of Romania. There was a census in Romania in 2011 that registered a large, and previously underestimated, decrease in population size. Evidently, the outmigration from Romania was underreported. Yet no rollback corrections were made, and Eurostat provides non-harmonized data for Romanian regions. Thus, we harmonized the population figures for Romanian regions.^2^

Finally, we excluded all non-European remote territories of France, Portugal, and Spain,^3^ which are outliers both in geographical and statistical terms.

The data set used for the analyses contains data for 263 NUTS-2 for the observed (2003–2012) and projected (2013–2042) periods.

### Measuring aging

We measure population aging as a decrease in the ratio of the working-age population to the non-working-age population. In line with Eurostat and UN definitions, we consider ages 15 and 65 as the margins of the working-age population. Thus, the measure of aging that we use is the ratio of population aged 15–64 to the population below 15 years of age and above 65. We call this indicator the total support ratio (TSR), which is in fact the inverse of the widely used total dependency ratio (UN Population Division 2002). There is some confusion around the use of the term support ratio in the literature. Quite often, children are not included in the calculation of the support ratio (Lutz 2006; Lutz et al. 2003; O’Neill et al. 2001). In that case, the indicator only shows the relative burden of the elderly population; UN Population Division (UN Population Division 2002) calls this indicator potential support ratio. In other papers, that deal not only with age structures of population but also with labor force participation and transfer accounts, by support ratio, authors usually mean the ratio of effective labor to effective consumers (Cutler et al. 1990; Lee and Mason 2010; Prskawetz and Sambt 2014). Another definition says that the support ratio is the size of the labor force as a share of the adult population (Börsch-Supan 2003). We prefer to explicitly call the ratio of the working-age to the non-working-age population the total support ratio, in line with the logic of the three versions of dependency ratio: total, youth, and old-age.

### Decomposition of growth in the total support ratio

To explain which demographic factors cause changes in the TSR, we apply a two-step decomposition. First, we examine to what extent changes in the TSR are due to changes in the size of the working-age population and to what extent to changes in the size of the non-working-age population. Second, we examine the demographic causes of changes in the working-age population.

At the *first step*, the overall change in the TSR is decomposed using the formula of Das Gupta (Das Gupta 1991):

1 $$ {\mathrm{TSR}}_2 - {\mathrm{TSR}}_1 = \frac{W_2}{{\mathrm{NW}}_2}-\frac{W_1}{{\mathrm{NW}}_1} = \left[\frac{1}{2}*\left({W}_2+{W}_1\right)*\left(\frac{1}{{\mathrm{NW}}_2}-\frac{1}{{\mathrm{NW}}_1}\right)\right]+\left[\frac{1}{2}*\left(\frac{1}{{\mathrm{NW}}_2}+\frac{1}{{\mathrm{NW}}_1}\right)*\left({W}_2-{W}_1\right)\right] $$

where $W$ is the working-age population, $\mathrm{N}\mathrm{W}$ is the non-working-age population, and subscripts $1$ and $2$ denote the beginning and the end of the period, respectively. The two right-hand side terms of Eq. 1 represent the effects of changes in non-working-age and working-age populations on the TSR, respectively. Note that changes in $W$ affect both the first and second terms, but the effect on the first term is very small compared with that on the second term. The average change in the first term due to the changes in the working-age population over all 263 regions was only −0.7% with a standard deviation of 3.3%.

At the *second step*, the working-age term in the second term of the right-hand side of Eq. 1 is decomposed further into changes due to the three components of the demographic balance at working ages: cohort turnover, migration, and mortality.

To estimate the components of change in working-age population, we use the demographic balance formula:

2 $$ {W}_2={W}_1+\mathrm{C}\mathrm{T}+{M}_W-{D}_W $$

where $\mathrm{C}\mathrm{T}$ is the cohort turnover between periods 1 and 2, ${M}_W$ is the net migration at working ages, and ${D}_W$ is the number of deaths at working ages. As the accuracy of migration records is always a problematic issue, following De Beer, Erf, and Huisman (2012), we derive net migration at working ages indirectly from Eq. 2 for the observed period, 2003–2012. For the projected period, 2013–2042, the migration data are provided by Eurostat, so we derive the numbers of deaths using the demographic balance formula. Cohort turnover is calculated as the difference between people entering working ages, aged 14, and people leaving working ages, aged 64.

Replacing the ${W}_2-{W}_1$ part of the working-age term in Eq. 1 using the demographic balance formula, Eq. 2, yields

3 $$ \frac{1}{2}*\left(\frac{1}{{\mathrm{NW}}_2}+\frac{1}{{\mathrm{NW}}_1}\right)*\left({W}_2-{W}_1\right)=\left[\frac{1}{2}*\left(\frac{1}{{\mathrm{NW}}_2}+\frac{1}{{\mathrm{NW}}_1}\right)*\mathrm{CT}\right] + \left[\frac{1}{2}*\left(\frac{1}{{\mathrm{NW}}_2}+\frac{1}{{\mathrm{NW}}_1}\right)*{M}_W\right]-\left[\frac{1}{2}*\left(\frac{1}{{\mathrm{NW}}_2}+\frac{1}{{\mathrm{NW}}_1}\right)*{D}_W\right] $$

The three right-hand side terms of Eq. 3 denote the effects of cohort turnover, migration at working ages, and mortality at working ages on TSR, respectively.

### Beta-convergence aproach to aging

To estimate beta convergence, we use the classical linear regression model specification, where change in a variable (in our case, total support ratio) over some period is regressed on the initial level. The specification looks as follows

4 $$ {\mathrm{TSR}}_2-{\mathrm{TSR}}_1 = \alpha + \beta\ {\mathrm{TSR}}_1 + \varepsilon $$

where $T S R$ is the total support ratio, $\alpha$ is the intercept of the regression line, $\beta$ is the regression coefficient, and $\varepsilon$ is the error term. If the regression coefficient is negative, then beta convergence is observed between years 1 and 2, meaning that the change in TSR is negatively correlated with the initial level of the TSR. Thus, beta convergence implies that a region with a relatively high TSR experiences less growth in the TSR than a region with a low TSR.

In convergence analysis, weights reflecting population sizes are often used (Dorius 2008; Goesling and Firebaugh 2004; Milanovic 2005; Theil 1989). Population-weighted convergence analysis shows whether inequality in the population becomes smaller; unit-weighted (in fact, non-weighted, as all units receive equal weights) convergence analysis tests whether the differences between units (countries/regions/districts) decrease. In this study, we are interested in the development of European regions as statistical units; thus, we choose the unit-weighted convergence analysis. Our choice is driven by the fact that European cohesion policy is aimed at regions, irrespective of their population sizes.^4^

The specification of the regression model allows to perform a decomposition of convergence (the beta coefficient) into various separate effects. To understand how each of the demographic factors contributed to beta convergence in aging, we decompose the dependent variable, the change in TSR (see the previous subsection), and run separate regressions for each partial change in TSR keeping the explanatory variable, the initial value of TSR, constant. A partial regression model shows the beta convergence of regions taking into account only the change in TSR due to the component under consideration. As the components of change in TSR add up to total change, and all the partial models have the same regressor, beta coefficients of the partial models add up to the total effect. That means, beta coefficients from convergence models for the change in TSR due to the dynamics of non-working-age population ($\mathrm{n}\mathrm{w}$) and working-age population ($w$) add up to the beta coefficient of the overall model ($g$), and beta coefficients from the models for cohort turnover ($\mathrm{c}\mathrm{t}$), migration at working ages ($\mathrm{mg}$), and mortality at working ages ($\mathrm{m}\mathrm{t}$) effects on TSR growth add up to beta coefficient from the model for the working-age population dynamics’ effect. For the ease of notation, we will refer to the partial model using the above symbols in brackets.

To use further the additive feature of the models, we ran a separate regression for each partial change in TSR in each year, dividing the study period into four decades—for each of the decades, the initial TSR distribution is used as an explanatory variable. The temporal decomposition gives insight into how the convergence process evolves throughout the study period. Summing up, in this paper, we use two dimensions of the decomposition of convergence in aging: demographic factors of the change in the TSR and time.

### Software

The analysis and the necessary data preparation were conducted using *R*, a language and environment for statistical computing, version 3.3.2 (R Core Team 2016). The crucial additional packages include *dplyr* (Wickham and Francois 2015), *tidyr* (Wickham 2016b), *ggplot2* (Wickham 2016a), *viridis* (Garnier 2016), and *rgdal* (Bivand et al. 2015). All the scripts are in the attachment for reproducibility.

## Results

### Descriptive results

**Fig. 1:**
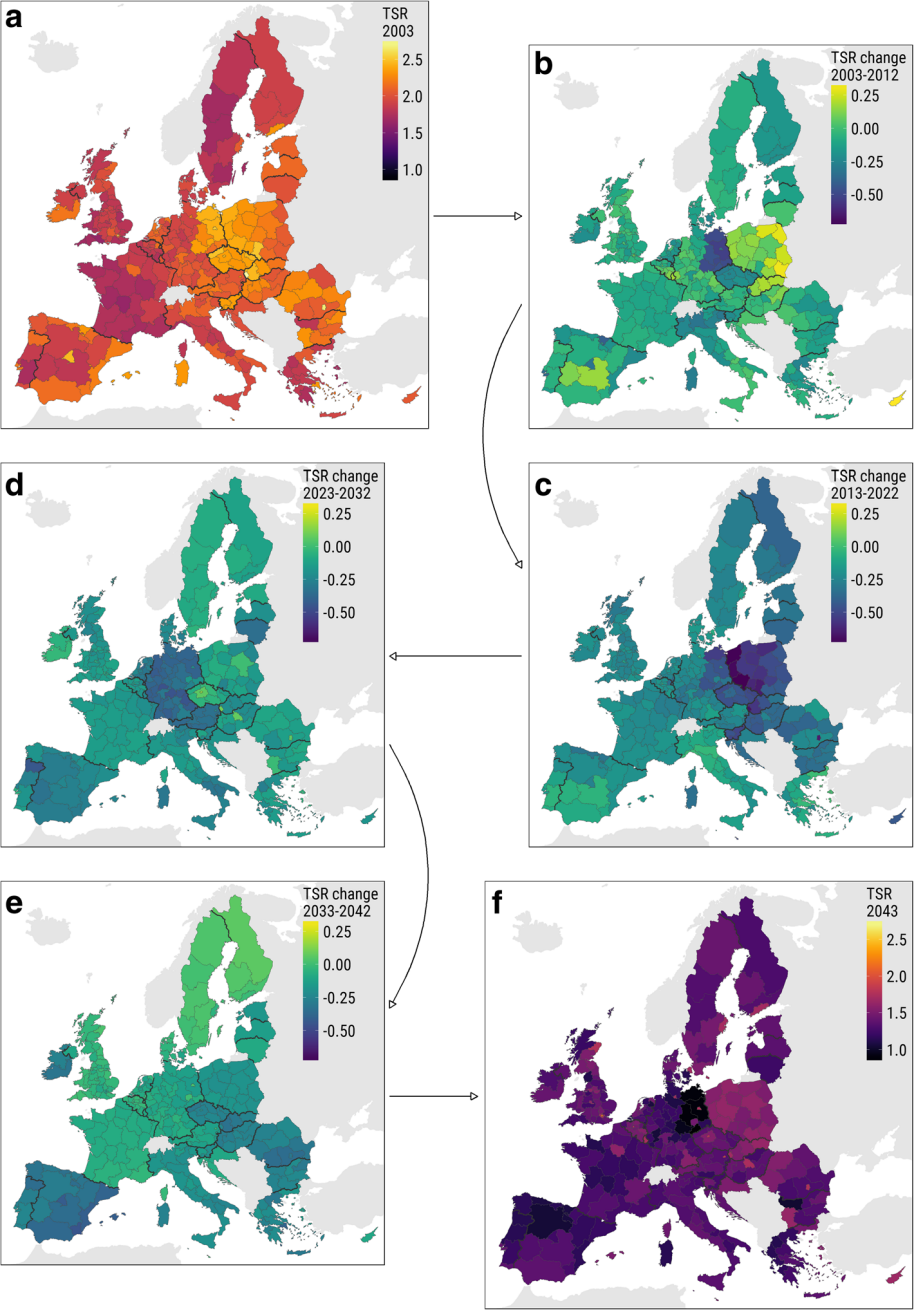
Total support ratio dynamics in the four decades between 2003 and 2043. *Notes*: **a** TSR in 2003. **b** TSR growth during the observed period, 2003–2012. **c**–**e** TSR growth in the three decades of the projected period, 2013–2022, 2023–2032, and 2033–2042, correspondingly. **f** TSR in 2043. Color scales are fixed for better comparison: (1) in maps **a** and **f** and (2) in maps **b**, **c**, **d**, and **e**

**Fig. 2:**
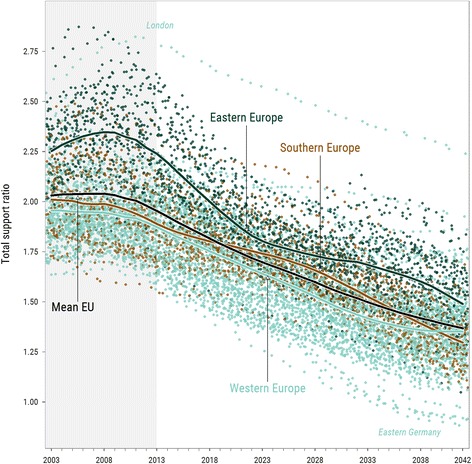
Total support ratio dynamics in Europe during the period 2003–2042, NUTS-2 regions, four subregions’ averages, and the European average. *Notes*: Each NUTS-2 region’s TSR value in each year of the study period is represented with a *point colored* according to EuroVoc definition of European subregions. *Lines* represent group averages. The most prominent outliers (London—*top*; and five regions of Easrtern Germany, excl. Berlin—*bottom*) are also labeled. Observed period marked with a *light-gray* background

The maps in Fig. 1 clearly reveal the story of a rapidly aging Europe. The first and the last maps show total support ratios of European NUTS-2 regions at the beginning and at the end of the whole study period, 2003 and 2043, 10 observed and 30 projected years; color scales are fixed for easier comparison. Virtually, every single region experiences a substantial decrease in the TSR over the study period; the average of all European regions decreased from 2.02 in 2003 to 1.96 in 2013 and is projected to further decrease to 1.37 by 2043, a 33% decrease over a period of 40 years (Fig. 2).

The spatial variation of the TSR across Europe is distinct both in the beginning and in the end of the study period. The spatial pattern seems very similar despite the 40 years of pronounced changes. Regions in Eastern Europe were relatively high in the initial distribution, and they are expected to remain in the top by the end of the study period: the dots in Fig. 2, colored according to the macro regions of Europe,^5^ show quite limited perturbation over time, and the lines showing the averages of subregions suggest the same. Even though the difference between East-European regions and the rest of Europe narrows, the distribution pattern changes only slightly.

The most prominent changes happen in the regions of Eastern Germany, a very special part of Europe in terms of demographic development (Klüsener and Goldstein 2016). Those regions were “closing the opportunities window” of demographic dividend at the beginning of the study period (Van Der Gaag and De Beer 2015). Thus, they experienced the biggest drop in the TSR during the first decade (Fig. 1b). With a usual decade-longtime lag, East-European regions are starting to experience a similar drop in the second decade of our study period (Fig. 1c). Yet, unlike Eastern Europe, the regions of Eastern Germany continue to descent from the top of the TSR distribution to the bottom. Quite a big decrease in the TSR happens in Southern Europe, especially in Spain, where the migration-driven temporary increase in the TSR is gradually changing towards a projected long-run decrease, which is mainly driven by population structure dynamics together with low fertility. The changes in the TSR over the four decades of the study period suggest that the east-west gradient in Europe is likely to change to a north-south gradient in the coming future.

The development of subregions’ average TSR over the study period (Fig. 2) demonstrates the cyclic effect of demographic waves, which is most evident for Eastern Europe but also visible for other two subregions—Southern and Western Europe. These demographic waves have a major effect on TSR and thus may considerably affect convergence in aging. The most interesting effect is the rapid TSR decrease that starts in 2010, when the large generation of European baby boomers started to cross the 65 years boundary (Reher 2015; Van Bavel and Reher 2013).

Some specific regions experience development that differs much from the other neighboring regions. For example, London, the biggest economic center in Europe, succeeds in constant attraction of relatively young population, which results in extremely high TSR (see the top path in Fig. 2 and also Figure 10 in Appendix). In contrast, regions of Eastern Germany experienced massive out-migration that, coupled with a strong shock of the lowest-low fertility in the recent past, results in a dramatic drop of TSR (see the bottom paths in Fig. 2 and Fig. 1).

### Decomposition of TSR growth

As described in the methodological part of the paper, the overall change in the TSR ($g$) can be decomposed into the effects of changes in the non-working-age population ($\mathrm{n}\mathrm{w}$) and the effects of changes in the working-age population ($w$). The latter can be further decomposed into the effects of cohort turnover ($\mathrm{c}\mathrm{t}$), migration at working ages ($\mathrm{mg}$), and mortality at working ages ($\mathrm{m}\mathrm{t}$).

**Fig. 3:**
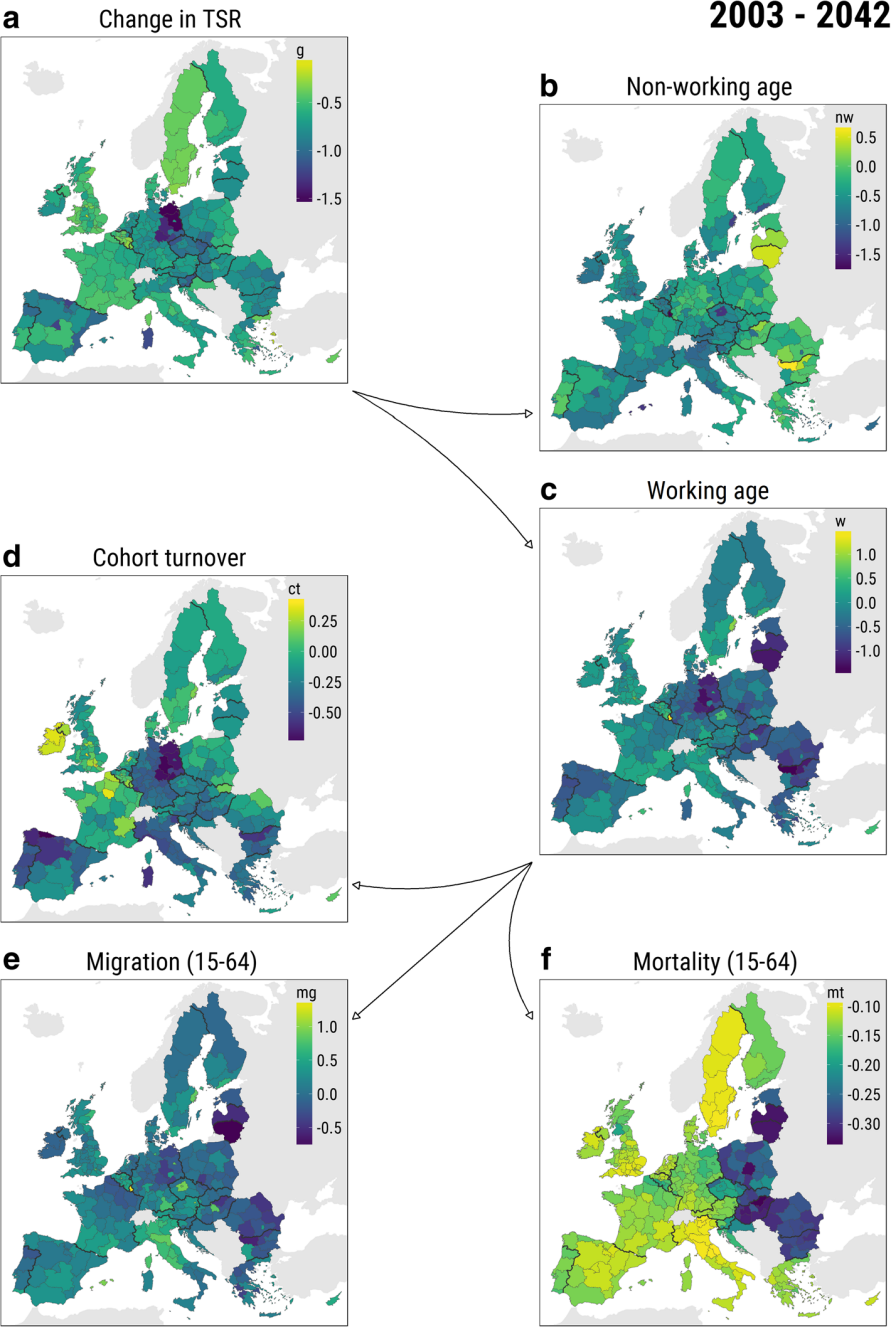
Decomposition of change in TSR between 2003 and 2043. *Notes*: **a** Overall change. **b** Change due to dynamics in non-working-age population. **c** Change due to dynamics in working-age population. **d** Change due to cohort turnover. **e** Change due to migration at working ages. **f** Change due to mortality at working ages. Color scales are panel specific due to the big difference in variables’ distributions

Figure 3 presents the two-step decomposition of change in the TSR during the whole study period (similar sets of maps for each of the decades can be found in the Appendix, Figures 6, 7, 8, and 9). Each of the partial effects reveals substantial variation across NUTS-2 regions, countries, and EuroVoc subregions. Not only the overall dynamics of the TSR are highly uneven but also the dynamics of each component.

The map of the overall change in the TSR (Fig. 3a) highlights the areas that faced the biggest absolute change. Eastern Germany experienced the most pronounced drop in the support ratio, with a considerable gap following Czech Republic, Slovenia, Spain, Northern Italy, Hungary, and Bulgaria. The biggest increase happened in Belgium (particularly, in Wallonia, the Southern part) and Luxembourg, Sweden, the UK, and Southern France.

The spatial variation in the TSR change due to the dynamics of non-working-age population (Fig. 3b) reveals two main findings. First, there is an evident north-south gradient, which can be explained by long-persisting European differences in fertility levels. Second, almost all major metropolitan regions are clearly visible because they experience a relatively sharp decline in the TSR due to the changes in the non-working-age population: Stockholm, Helsinki, Copenhagen, London, Amsterdam, Berlin, Prague, Budapest, Bucharest, Vienna, Paris, Rome, and Madrid. Evidently, population replacement in the metropolitan areas is mainly driven by migration (Fig. 3e), rather than cohort turnover. The spatial variation of the TSR growth due to the changes in working-age population (Fig. 3c) clearly shows the attractiveness of the regions for the labor force.

**Fig. 4:**
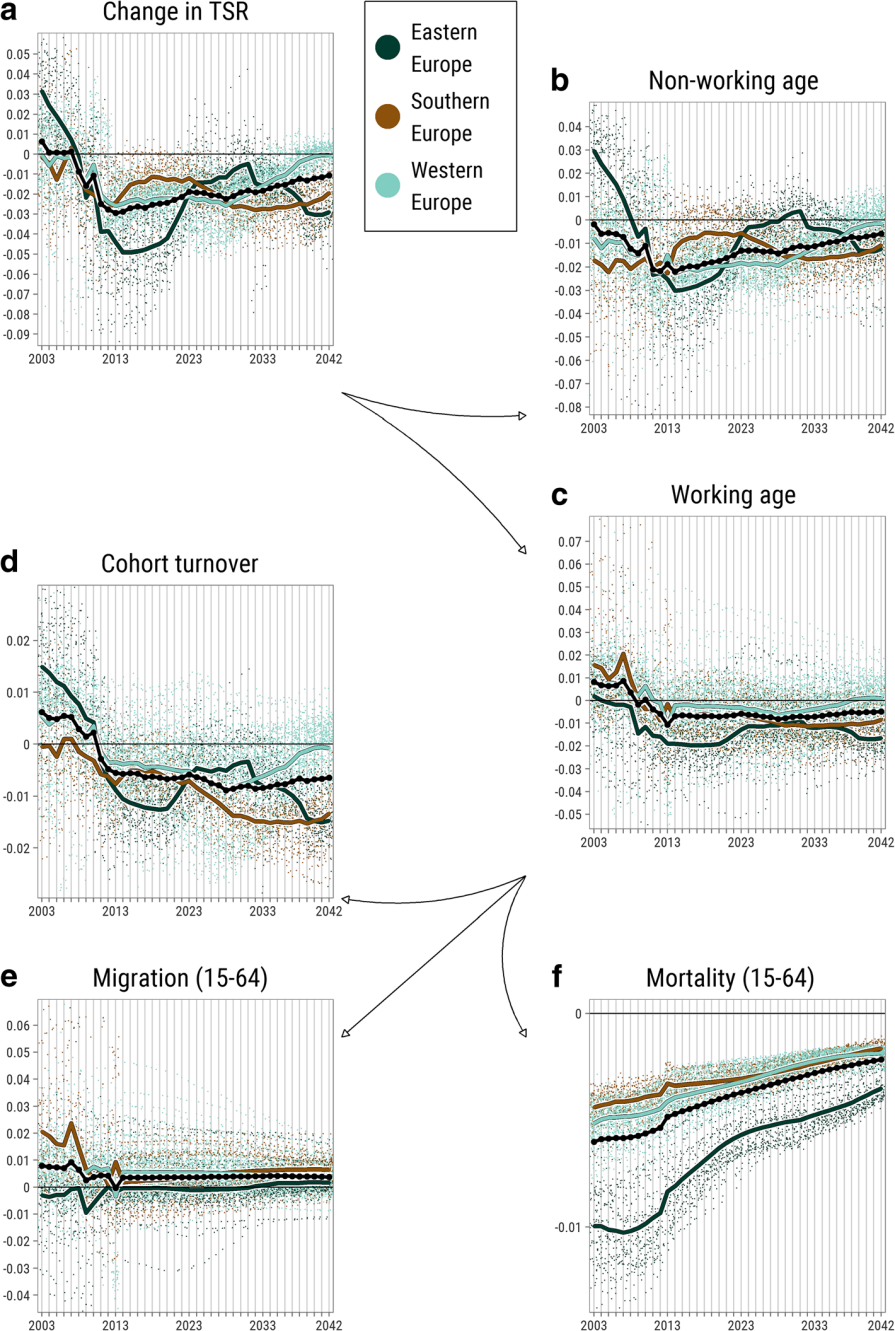
**a**–**f** Distributions of the decomposed components of change in TSR, all years between 2003 and 2043. *Notes*: Each NUTS-2 region is represented with a *point colored* according to EuroVoc definition of European subregion. Scales on *y*-axes are panel specific due to the big difference in variables’ distributions

The spatial pattern of changes in the TSR due to cohort turnover (Fig. 3d) is distinctively similar to what we know about fertility (Frejka and Sobotka 2008) and child migration levels in Europe (Wilson et al. 2013). Interestingly, lots of metropolitan areas have relatively higher increase or lower decrease in the TSR due to cohort turnover, which, probably, means that quite often, people leave these areas before turning 65 (see, for example, the development of the population pyramid of London in the Appendix, Figure 10). The effect of migration at working ages on the TSR (Fig. 3e), apart from the mentioned above metropolitan areas regularity, shows some east-west gradient: emigration of working-age population from East-European regions, and especially from Baltic countries, is particularly high. But the most pronounced east-west gradient appears at the map of mortality at the working ages component of the change in the TSR (Fig. 3f). The prevalence of mortality at ages between 15 and 64 in the regions of Eastern Europe is striking. Even the optimistic convergence-based scenarios of Eurostat population projection do not promise that this divide would vanish in the coming three decades (Fig. 4f).

Figure 4 illustrates the importance of demographic waves in the development of population structures. This is particularly evident for East-European regions. The downfall of fertility in the 1990s produced a very small generation giving a short-term alleviating effect (demographic dividend), but resulting in a big negative impact of cohort turnover on the TSR 15 years later and a smaller alleviating echo effect about 30 years later. The timing of the effect of migration on the change in the TSR is only visible in the observed part of the study period. The pre- and post-2008 economic crisis migration shocks are very pronounced (note also that the *y*-axis scale range is relatively big for the migration component). In the projected part of the study period, according to the converging baseline assumption, migration intensities are extrapolated with reducing variance, which result in a very smooth development of an almost fixed distribution. With such a projection, migration at working ages can hardly have any effect on convergence in aging (see the next subsection).

### Beta-convergence analysis

**Fig. 5:**
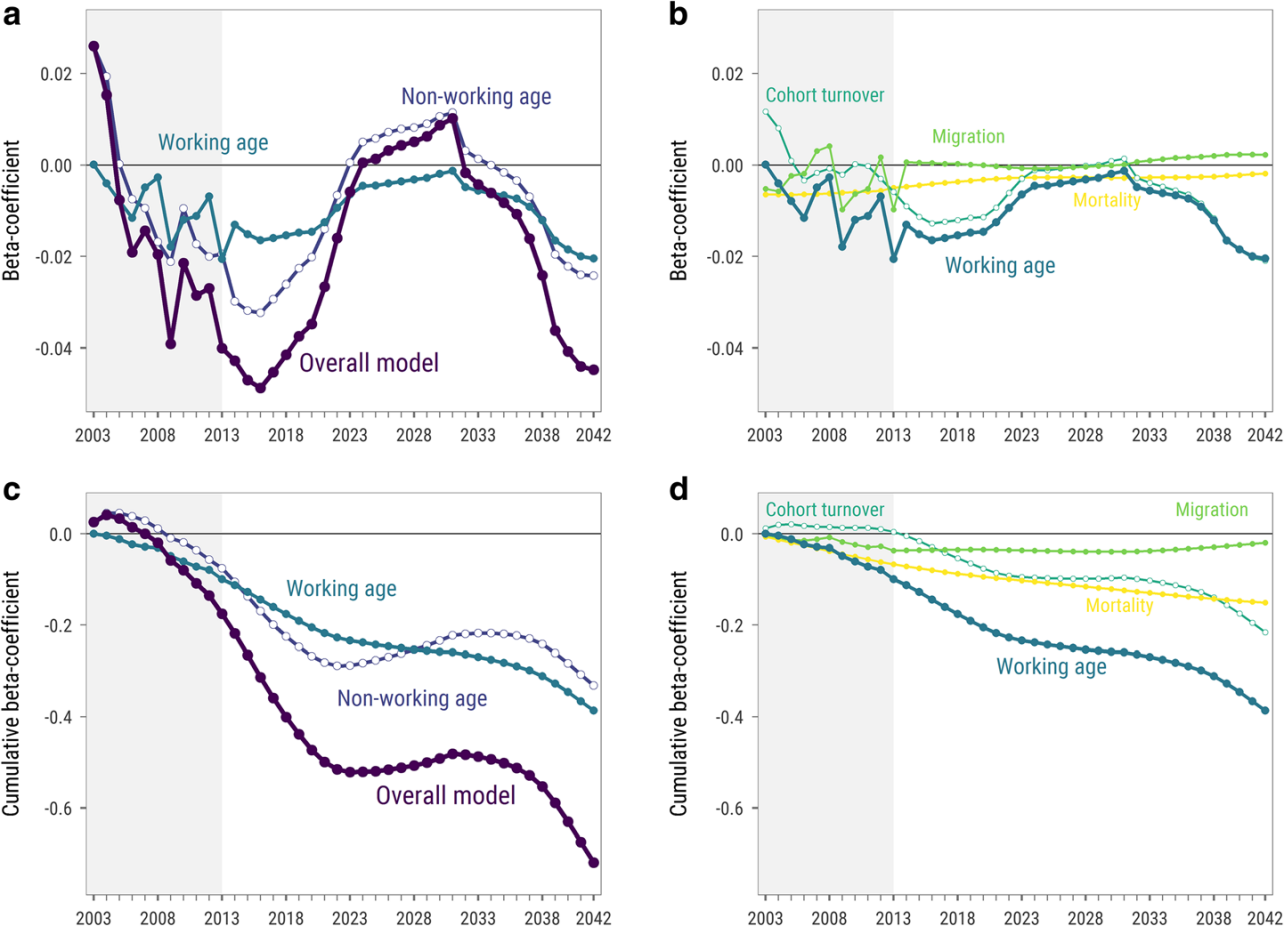
Bi-dimensional decomposition of beta-convergence estimates by (1) component of change in TSR and (2) time. *Notes*: *Each point* represents beta coefficient from the corresponding partial model. The *left panels* show the first step of TSR growth decomposition; the *right panels* shows the second step. Panels **a** and **b** show beta-convergence estimates for each year separately; panels **c** and **d** show the cumulative effect. Observed period marked with a *light-gray* background

**Fig. 6:**
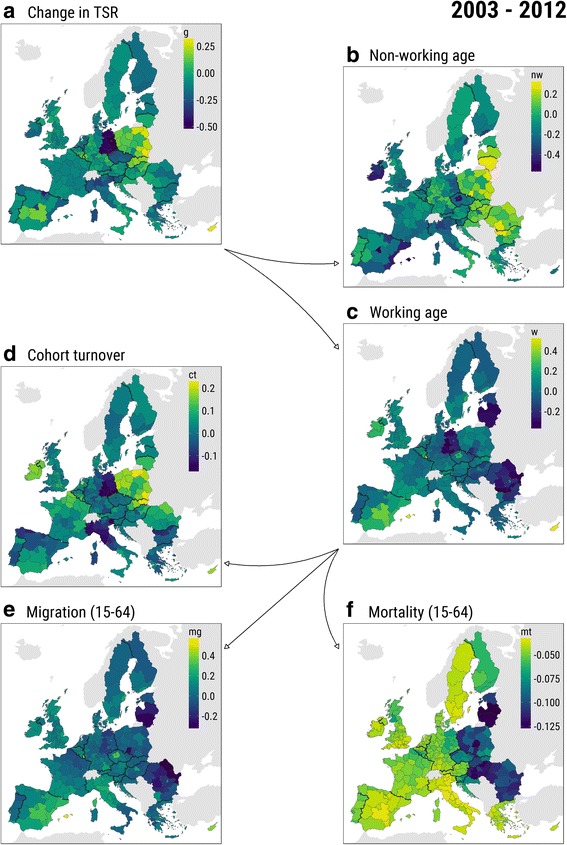
Decomposition of change in TSR between 2003 and 2013. *Notes*: **a** Overall change. **b** Change due to dynamics in non-working-age population. **c** Change due to dynamics in working-age population. **d** Change due to cohort turnover. **e** Change due to migration at working ages. **f** Change due to mortality at working ages. Color scales are panel specific due to the big difference in variables’ distributions

**Fig. 7:**
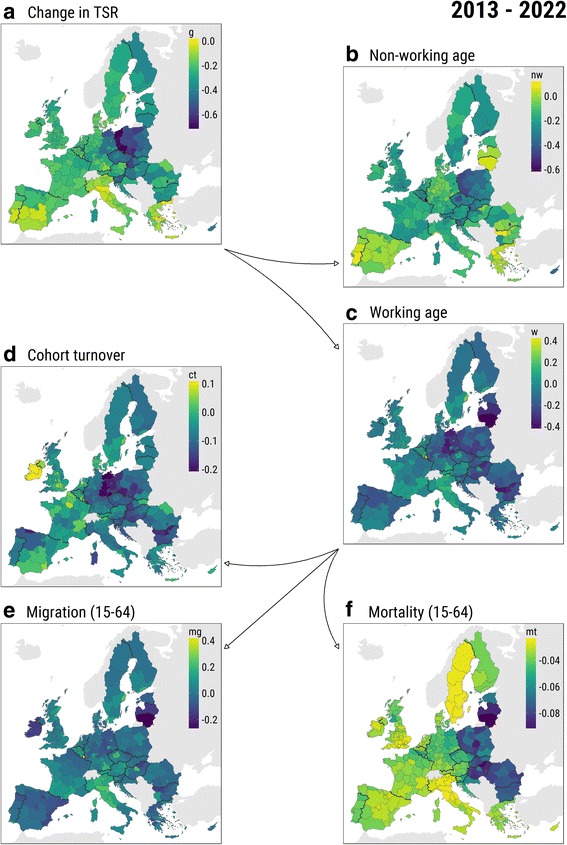
Decomposition of change in TSR between 2013 and 2023. *Notes*: **a** Overall change. **b** Change due to dynamics in non-working-age population. **c** Change due to dynamics in working-age population. **d** Change due to cohort turnover. **e** Change due to migration at working ages. **f** Change due to mortality at working ages. Color scales are panel specific due to the big difference in variables’ distributions

**Fig. 8:**
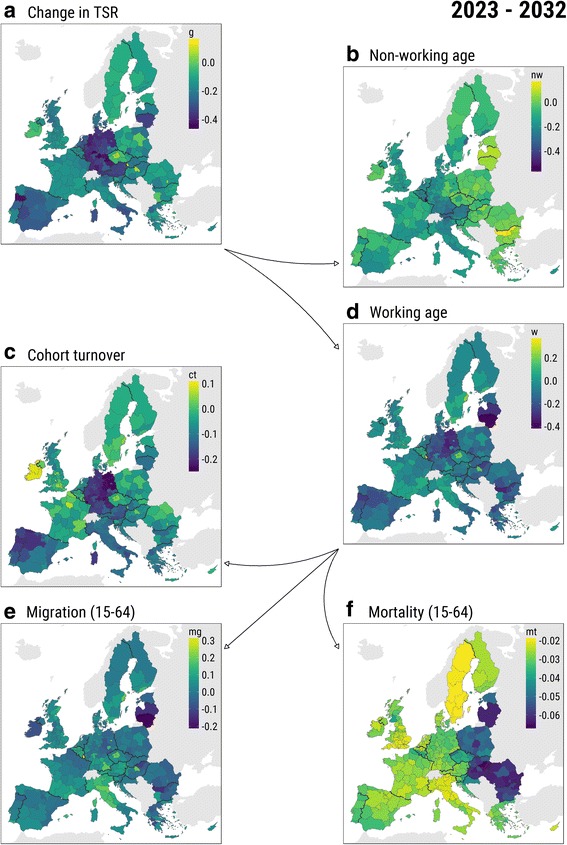
Decomposition of change in TSR between 2023 and 2033. *Notes*: **a** Overall change. **b** Change due to dynamics in non-working-age population. **c** Change due to dynamics in working-age population. **d** Change due to cohort turnover. **e** Change due to migration at working ages. **f** Change due to mortality at working ages. Color scales are panel specific due to the big difference in variables’ distributions

**Fig. 9:**
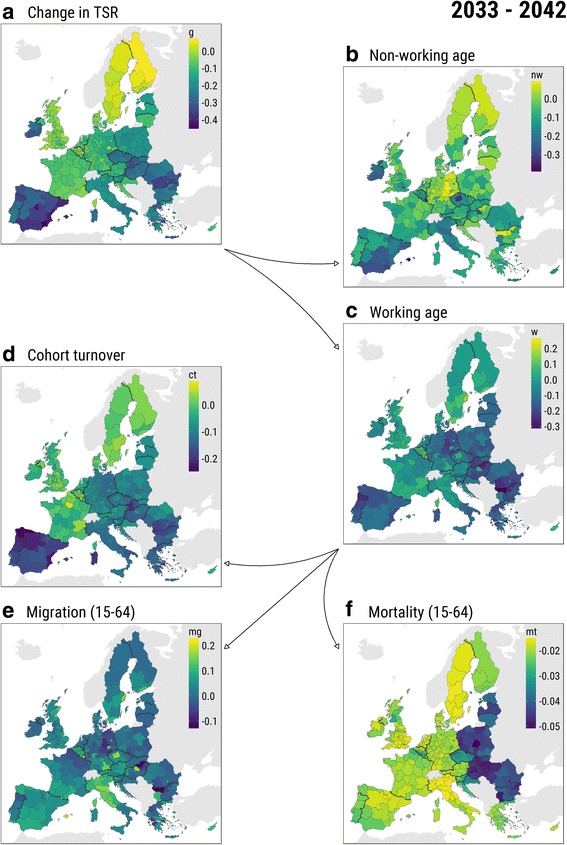
Decomposition of change in TSR between 2033 and 2043. *Notes*: **a** Overall change. **b** Change due to dynamics in non-working-age population. **c** Change due to dynamics in working-age population. **d** Change due to cohort turnover. **e** Change due to migration at working ages. **f** Change due to mortality at working ages. Color scales are panel specific due to the big difference in variables’ distributions

**Fig. 10:**
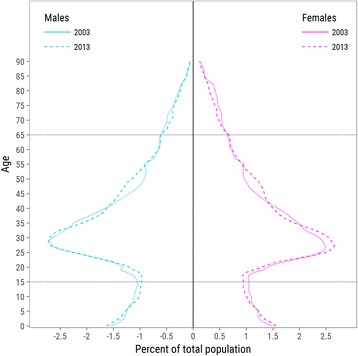
Population pyramids of London in 2003 and 2013

The results of the beta-convergence modeling for all regions of Europe are shown in Fig. 5; panels A and B show the components of the first and the second steps of the decomposition of changes in the TSR, respectively. Each point in the plot represents an estimate of the beta coefficient from the corresponding partial model. Panels C and D show the same model estimates but in a cumulative way, revealing the overall convergence process throughout the study period.

**Fig. 11:**
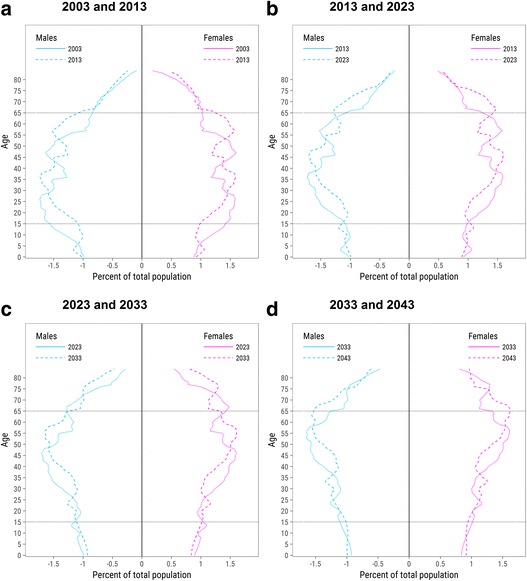
**a**–**d** Population pyramids of Eastern Europe in 2003, 2013, 2023, 2033, and 2043

The dynamics of beta coefficients from $g$ models indicate that there was divergence (positive beta coefficients) in 2003 and 2004 and then convergence (negative beta coefficients) for the rest of the observed period with local peaks in 2009 and 2013. The rapid convergence continues till the beginning of the 2020s. From two previous results subsections, we know that this period is characterized by the anticipated rapid decrease in the TSR in East-European regions. Then, there is hardly any convergence in the 2020s and early 2030s while East-European regions experience an alleviating echo effect of a relatively smaller generation born to the very small generation of parents born in the 1990s (see, for example, population pyramids for Romania in the Appendix, Figure 11). Finally, fast convergence starts again in the middle of the 2030s when the smaller “echo generation” enters working ages. In short, most of the regional convergence in aging in Europe seems to be driven by the dynamics of the TSR in the regions of Eastern Europe.

Thus, the overall convergence trend is mainly set by changes in the size of the non-working-age population in the first half of the study period; changes in the size of the working-age population contribute much less to the overall convergence. Though, in the second half of the study period, convergence is mainly driven by the working-age population. In the end, the cumulative contributions of both components are almost equal.

The contribution of $\mathrm{c}\mathrm{t}$ is very similar to the effect of $\mathrm{n}\mathrm{w}$: it contributes to divergence slightly in the beginning of the period and then follows closely the population structure dynamics in Eastern Europe. The impact of $\mathrm{mg}$is quite insignificant throughout the study period due to the mentioned above features of Eurostat regional population projection. The influence of $\mathrm{m}\mathrm{t}$ is the most stable, which can be explained by the very slow pace of changes in mortality rates and the huge initial differences between Eastern Europe and the rest. It contributes to convergence because both the initial TSR and mortality rates at working ages are higher in East-European regions. By the end of the study period, the cumulative effect of the moderate but stable year-by-year $\mathrm{m}\mathrm{t}$ contributions accounts for about 40% of the convergence in $w$.

## Conclusion and discussion

In this paper, we investigate how regional differences in population aging across Europe developed over the last decade and how they are likely to evolve in the coming three decades. The results show that there was convergence in aging during the biggest part of the period 2003–2012 and it is anticipated during the first and the third decades of the projected period (2013–2022 and 2033–2042). Note that the occurrence of convergence in the future depends on the accuracy of the Eurostat projections. These projections depend on assumptions about future changes in cohort turnover, mortality, and migration. While assumptions about cohort turnover and mortality generally are reliable, the validity of assumptions about future migration is rather uncertain.

The speed of convergence depends mainly on the development of the total support ratio in East-European regions in relation to the rest of Europe. Convergence is, by definition, a temporary process. The convergence in aging among European NUTS-2 regions throughout the 40 years long study period can be explained by the fact that the initial variation in aging was at a local peak because of the East-European regions that experienced the ending phase of the window of demographic opportunities.

Population structures affect convergence in aging through cohort turnover and changes in the size of the non-working-age population. Growth of the non-working-age population is responsible for approximately half of the overall convergence in the study period. Of the second half, which is attributed to the effect of growth in the working-age population, cohort turnover is responsible for about 60% of the effect.

Mortality at working ages has the most stable impact on convergence in aging. It accounts for about 40% of the convergence effect through changes in the size of the working-age population. Interesting in itself, this finding limits the scope for policy options: if policy makers aim at convergence in mortality, this may be in conflict with aiming at convergence in population aging. Even though convergence in aging may be desirable, the persisting higher mortality in East-European regions is, by no means, a policy option. Yet this component is likely to contribute significantly to convergence in aging in the coming decades because improvements in mortality rates go very slowly (Vallin and Meslé 2004).

Quite surprisingly, migration at working ages assumptions in the Eurostat projections has an almost no effect on convergence in aging in the long run. This can be explained by the assumption that there will be convergence in every demographic indicator, which are baseline assumptions of the EUROPOP2013 regional population projections. Interestingly, the contribution of migration at working ages is crucial in explaining the biggest fluctuation of the effect of change in working-age population during the observed period. The most notable is the change of the trend in 2009, which is likely to be caused by sharpened out-migration from East-European regions after the outbreak of the economic crisis, and the preceding local peak of 2004–2005 was, most likely, linked to the increased migration intensities after the biggest EU enlargement. The relative importance of migration during the observed period and the lack of effect on the projected period indicate that convergence in migration flows, as projected by the basic Eurostat scenario, may not be the most realistic outcome.

The relatively big impact of cohort turnover leaves room for policy options, since the size of the impact depends on the age boundaries of 15 and 65 years. If policies aimed at raising the retirement age will be effective, the upper age boundary of 65 should be raised. This will have a positive impact on the level of the TSR. Note that crossing the age margin of 65 may have different implications for different parts of Europe due to varying participation rates after 65 (Sanderson and Scherbov 2010, 2015; Sanderson and Scherbov 2007). Similarly, with the persistent growth of educational attainment, the lower border of working ages may be raised (Harper 2014). This will have a negative impact on the level of the TSR. In this paper, we focused on the pure demographic effects that alter population structures, but the societal meaning of age is not constant. Thus, the use of more nuanced definitions of dependent populations (Spijker and MacInnes 2013) and labor support (Prskawetz and Sambt 2014) are welcome in the further research on regional convergence in population aging in Europe.

One important question is whether convergence in population aging contributes to economic convergence. Although researchers mainly find proofs of the negative effects of accelerating aging on the economy and on social structures, some demographers call for a calmer evaluation of the consequences of aging (Lloyd-Sherlock et al. 2012; Van Dalen and Henkens 2011; Vaupel and Loichinger 2006). Moreover, some economists even doubt the negative influence of population aging on economic development, at least in the beginning of the period of accelerated aging (Gómez and De Cos 2008). But even if we rely on a negative link between aging and economic development, the interplay between convergence in aging and economic cohesion is not stable over time and space: it depends on the change in productivity and labor force participation (Kashnitsky et al. 2017).

The mentioned limitations ask for further research on convergence in aging. In this paper, we analyzed for the first time the evolution of population structures using beta-convergence modeling and attempted to understand how demographic components of population growth contribute to the convergence process. Our results together with theoretical aspirations and prior research in the field (De Beer et al. 2012) indicate that examining urban/rural differences will be very useful for the analysis of convergence in aging.

## Appendix

**Table 1:** Summary statistics for the observed period by countries

Country	# of NUTS-2 reg.	Total population				TSR	Average TSR growth due to						TSR
		Country	Mean pop. of region	Country	Mean pop. of region		*g*	nw	w	mt	mg	ct	
		2003	2003	2013	2013	2003	2003–2012						2013
Eastern Europe													
BG	6	7.8	1.3	7.3	1.2	2.14	−0.126	0.090	−0.216	−0.109	−0.061	−0.046	2.02
CZ	8	10.2	1.3	10.5	1.3	2.40	−0.235	−0.241	0.006	−0.084	0.099	−0.009	2.16
EE	1	1.4	1.4	1.3	1.3	2.10	−0.135	−0.005	−0.130	−0.104	−0.064	0.038	1.97
HU	7	10.1	1.4	9.9	1.4	2.17	−0.002	0.077	−0.079	−0.117	0.009	0.029	2.17
LT	1	3.4	3.4	3.0	3.0	2.02	0.020	0.309	−0.289	−0.125	−0.278	0.114	2.04
LV	1	2.3	2.3	2.0	2.0	2.13	−0.124	0.187	−0.310	−0.129	−0.214	0.032	2.01
PL	16	38.2	2.4	38.1	2.4	2.26	0.151	0.130	0.021	−0.098	−0.040	0.159	2.44
RO	8	21.8	2.7	20.0	2.5	2.24	−0.075	0.120	−0.194	−0.105	−0.168	0.078	2.17
SK	4	5.4	1.3	5.4	1.4	2.44	0.069	0.023	0.046	−0.097	0.007	0.137	2.51
Southern Europe													
CY	1	0.7	0.7	0.9	0.9	2.05	0.327	−0.214	0.541	−0.039	0.404	0.176	2.38
EL	13	11.0	0.8	11.1	0.9	1.91	−0.116	−0.106	−0.010	−0.041	0.037	−0.005	1.79
ES	19	39.9	2.5	44.5	2.8	2.13	−0.123	−0.304	0.180	−0.042	0.226	−0.004	2.00
HR	2	4.3	2.2	4.3	2.1	2.01	0.005	0.008	−0.003	−0.071	0.054	0.014	2.01
IT	21	57.1	2.7	59.7	2.8	1.98	−0.144	−0.165	0.021	−0.037	0.115	−0.056	1.84
MT	1	0.4	0.4	0.4	0.4	2.17	−0.016	−0.143	0.128	−0.046	0.099	0.074	2.15
PT	7	10.0	2.0	10.0	2.0	1.98	−0.130	−0.110	−0.019	−0.052	0.048	−0.015	1.85
SI	2	2.0	1.0	2.1	1.0	2.36	−0.195	−0.211	0.016	−0.068	0.073	0.011	2.17
Western Europe													
AT	9	8.1	0.9	8.5	0.9	2.12	−0.051	−0.115	0.064	−0.048	0.086	0.026	2.07
BE	11	10.4	0.9	11.2	1.0	1.90	0.000	−0.151	0.151	−0.051	0.137	0.065	1.90
DE	38	82.5	2.2	82.0	2.2	2.07	−0.116	−0.054	−0.062	−0.051	0.029	−0.041	1.95
DK	5	5.4	1.1	5.6	1.1	1.95	−0.143	−0.158	0.015	−0.049	0.045	0.020	1.81
FI	5	5.2	1.0	5.4	1.1	1.99	−0.161	−0.202	0.041	−0.055	0.074	0.022	1.83
FR	26	60.1	2.7	63.7	2.9	1.81	−0.088	−0.150	0.062	−0.049	0.044	0.066	1.73
IE	2	4.0	2.0	4.6	2.3	2.05	−0.165	−0.427	0.262	−0.040	0.140	0.162	1.89
LU	1	0.4	0.4	0.5	0.5	2.04	0.187	−0.258	0.445	−0.048	0.390	0.102	2.23
NL	12	16.2	1.3	16.8	1.4	2.07	−0.152	−0.174	0.022	−0.045	0.017	0.050	1.92
SE	8	8.9	1.1	9.6	1.2	1.81	−0.070	−0.132	0.062	−0.038	0.080	0.020	1.74
UK	37	59.5	1.6	63.9	1.7	1.88	−0.043	−0.154	0.111	−0.046	0.107	0.050	1.84

## Footnotes

1 Currently (as of 2017), European Union consists of 28 countries, which are the following: Austria, Belgium, Bulgaria, Croatia, Cyprus, Czech Republic, Denmark, Estonia, Finland, France, Germany, Greece, Hungary, Ireland, Italy, Latvia, Lithuania, Luxembourg, Malta, The Netherlands, Poland, Portugal, Romania, Slovakia, Slovenia, Spain, Sweden, and UK.

2 Using 2003 population structure as the reference and the mortality data, we estimate cohort-wise the anticipated population structure in 2012 with an assumption of no migration. The difference between the estimated and the observed population is explained by migration. While harmonizing the data, we kept the observed migration trends and distributed the excessive migration evenly across all the years of observation before 2012.

3 The excluded NUTS-2 regions are the following: ES63, ES64, ES70, FR91, FR92, FR93, FR94, PT20, and PT30.

4 One of the objectives of NUTS was to provide more or less comparable administrative division for all countries of Europe. Nevertheless, in 2013, population figures for single NUTS 2 regions ranged from 28.5 thousands in Aland island (Finland) to almost 12 million in Ile-de-France (Paris and surroundings, France).

5 We divide Europe into three subregions: eastern, southern, and western. Initially, we tried to use the official subdivision of European countries into northern, western, southern and eastern parts (EuroVoc 2015). But the subset of northern regions turned out to be too small and heterogeneous. So we merged Scandinavia with Western Europe and Baltic regions—with Eastern Europe.
